# Comparative Efficacy and Safety of Direct Oral Anticoagulants Versus Warfarin in Atrial Fibrillation Patients with Chronic Liver Disease: A Systematic Review and Meta-analysis

**DOI:** 10.19102/icrm.2024.15103

**Published:** 2024-10-15

**Authors:** Syed Muhammad IbnE Ali Jaffari, Fnu Karishma, Syeda Urooba Shah, Robish Kishore, Avinash Kumar, Fnu Kajal, Maira Khalid, Avesh Kumar, Huda Anum, Zarmina Ali, Rimsha Irfan, Muhammad Ahsan Naseer Khan, Abdul Rehman Saleem, Hamza Islam, Rabia Islam

**Affiliations:** 1 Department of Medicine, Shalamar Medical and Dental College, Lahore, Pakistan; 2 Department of Medicine, Ghulam Muhammad Mahar Medical College, Sukkur, Pakistan; 3 Department of Medicine, Jinnah Sindh Medical University, Karachi, Pakistan; 4 Department of Medicine, Bahria University Medical and Dental College, Karachi, Pakistan; 5 Department of Medicine, Indus Hospital, Karachi, Pakistan; 6 Department of Medicine, King Edward Medical University, Lahore, Pakistan; 7 Department of Medicine, Shaheed Mohtarma Benazir Bhutto Medical College, Karachi, Pakistan; 8 Department of Medicine, Punjab Medical College, Faisalabad, Pakistan

**Keywords:** Atrial fibrillation, chronic liver disease, direct oral anticoagulants, DOACs, meta-analysis, warfarin

## Abstract

Atrial fibrillation (AF) is a prevalent cardiac arrhythmia. Direct oral anticoagulants (DOACs), with superior efficacy and safety, have emerged as a promising alternative to warfarin. This systematic review and meta-analysis aimed to compare the safety and efficacy of DOACs and warfarin in patients with AF and chronic liver disease (CLD). A systematic search was undertaken in PubMed, the Cochrane Library, and Google Scholar to identify studies comparing the effectiveness of DOACs and warfarin in patients diagnosed with AF and CLD. Subsequent analyses were carried out using the random-effects model. This meta-analysis included eight studies involving 20,684 participants; baseline characteristics indicated a prevalent male presence (56.7%), with an average age of 61.63 ± 9 years. Primary outcomes demonstrated that DOACs were associated with significantly reduced all-cause mortality (relative risk [RR], 0.73; 95% confidence interval [CI], 0.56–0.95; *I*^2^ = 84%; *P* = .02) and ischemic stroke risk (RR, 0.62; 95% CI, 0.45–0.86; *I*^2^ = 61%; *P* = .004). Secondary outcomes revealed a significantly reduced risk of major bleeding with DOACs compared to warfarin, while gastrointestinal bleeding showed a non-significant decrease. Intracranial hemorrhage risk was significantly lower with DOACs compared to warfarin. DOACs demonstrate superior safety and efficacy compared to warfarin, evidenced by reduced rates of all-cause death, ischemic stroke, severe bleeding, and cerebral hemorrhage. Further randomized controlled trials are essential to enhance the evidence base for DOACs across diverse patient populations.

## Introduction

Atrial fibrillation (AF) is the most common cardiac arrhythmia among the general population. Numerous chronic illnesses, such as obesity, diabetes, arterial hypertension (HTN), thyroid dysfunction, and inflammatory diseases, have been identified as risk factors for AF.^1^ People with chronic liver disease (CLD) and a normal heart structure may be more susceptible to AF. This is due to the fact that CLD can produce inflammatory substances in the blood and disrupt the autonomic nervous system, leading to conditions that contribute to arrhythmias such as AF.^2^ Several other factors are also suggested to contribute to the association between AF and CLD, which include issues such as dyslipidemia, increased insulin resistance, and renin–angiotensin system activation.^3^ Also, CLD has been attributed to an increased incidence of both thromboembolic and bleeding events in people with AF.^4^ Furthermore, recent studies have shown that CLD is associated with an increased risk of severe cardiovascular events and all-cause death. These data suggest that, in patients with AF, the presence of CLD significantly increases the risk of stroke and other severe cardiovascular events.^4,5^ The development of direct oral anticoagulants (DOACs) has resulted in considerable advances in preventing these challenges. Compared to warfarin, DOACs are preferable due to their greater efficacy and safety profile, predictable effects that do not require routine monitoring, and reduced interactions.^5^ The availability of DOACs is expected to provide significant benefits by increasing the appropriate use of anticoagulants and, as a result, leading to better stroke prevention in patients with CLD and AF.^5^ However, while the use of DOACs in patients with AF and CLD is appealing, it is important to remember their higher cost and the need for precautions in cases of severe liver damage.^6,7^ Few studies have been conducted on the effectiveness and safety of DOACs compared to warfarin in individuals with CLD and AF who require anticoagulant medication. To the best of our understanding, we have performed a first systematic review and meta-analysis on this subject matter, encompassing the entire body of literature, in order to contrast the safety and efficacy profiles of DOACs and warfarin in patients with CLD and AF.

## Methods

This meta-analysis adhered to the principles outlined in the Preferred Reporting Items for Systematic Reviews and Meta-Analyses (PRISMA) statement,^8^ aiming to ensure methodological transparency and bolster the overall reliability of the study.

### Data sources and search strategy

A systematic and thorough search strategy was employed to identify relevant studies. Electronic databases, including PubMed, the Cochrane Library, and Google Scholar, were explored until February 2023, without language restrictions. The search used a combination of Medical Subject Heading terms and keywords, encompassing terms such as “direct oral anticoagulants,” “DOACs,” “warfarin,” “atrial flutter,” “liver cirrhosis,” and others as detailed in **Supplementary Table S1**. Two independent researchers conducted the literature search to avoid selection bias. Discrepancies were resolved through consensus, with a third researcher consulted if needed.

### Study selection

A rigorous approach was undertaken to select studies for the meta-analysis. Two independent reviewers carefully examined titles and abstracts and then evaluated full texts. Discrepancies in study inclusion were addressed through extensive discussion and, when necessary, consultation with a third reviewer.

Inclusion criteria included the following:

- Adult patients (≥18 years of age) diagnosed with AF and documented evidence of CLD, including cirrhosis
- Studies comparing the use of DOACs, including dabigatran, rivaroxaban, apixaban, and edoxaban, with warfarin or coumadin
- Randomized controlled trials (RCTs), prospective and retrospective cohort studies, and observational studies with a well-defined control group
- Studies reporting both effectiveness and safety outcomes of anticoagulant therapy
- Studies with a follow-up duration of ≥6 months to capture short-term and long-term outcomes

Exclusion criteria included the following:

- Pediatric populations (<18 years of age)
- Patients with non-AF indications for anticoagulation
- Patients with AF but not diagnosed with CLD
- Studies assessing anticoagulant therapies other than DOACs or warfarin
- Studies not comparing DOACs with warfarin
- Case reports, case series, letters, comments, and review articles
- Studies not reporting relevant effectiveness and safety outcomes
- Studies with a follow-up duration of <6 months
- Studies conducted before 2000, as older data may not reflect current clinical practice standards

### Data extraction

Two evaluators independently conducted data extraction using a standardized form, encompassing study characteristics (eg, author, publication year, study design), participant demographics, mean CHA_2_DS_2_-VASc_2_ score, mean HAS-BLED score, comorbidities, and any other drugs that the patients were taking along with anticoagulants. Additionally, outcomes, including all-cause mortality, ischemic stroke, major bleeding, gastrointestinal (GI) bleeding, and intracranial hemorrhage (ICH), were extracted. These outcomes were categorized into primary outcomes, composed of all-cause mortality and ischemic stroke, and secondary outcomes encompassing major bleeding, GI bleeding, and ICH. Discrepancies were resolved through discussion or by involving a third reviewer when necessary.

### Quality assessment

Two reviewers independently evaluated the methodological quality and risk of bias using specific tools, including the Cochrane risk of bias tool^9^ for RCTs and the Newcastle–Ottawa Scale^10^ for observational studies. The assessment focused on study design, participant selection, blinding, outcome reporting, and other parameters.

### Data analysis

The statistical analysis for this meta-analysis was carried out using Review Manager (RevMan) version 5.4.1, a tool developed via collaboration between the Nordic Cochrane Centre and the Cochrane Collaboration in Denmark in 2014. The analysis exclusively included comparative studies, and the outcomes were presented through forest plots, illustrating the combined effect of relative risks (RRs) for dichotomous results and weighted mean differences for continuous outcomes. A random-effects model with generic-inverse variance was employed to enhance results accuracy.

To ascertain significance, *P* < .05 was considered. Funnel plots were generated for primary outcomes to assess the potential for publication bias. The degree of heterogeneity, determined by Higgin’s *I*^2^ test,^11^ was categorized as low, moderate, or high. In cases of substantial heterogeneity (>75%), a sensitivity analysis was performed, systematically excluding one study at a time to discern individual studies’ impact on the overall findings.

It is essential to note that ethical approval was deemed unnecessary for this meta-analysis as it involved the synthesis and analysis of previously published data without direct engagement with human subjects. All data were sourced from publicly available materials, ensuring compliance with ethical guidelines and maintaining the confidentiality of patient information.

## Results

### Eligible studies

**Figure 1:**
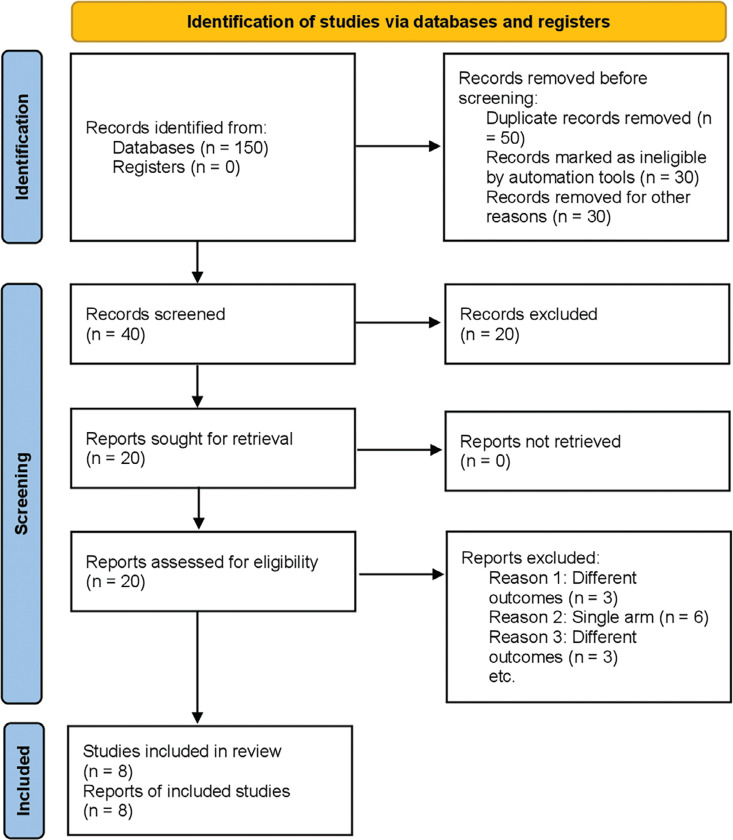
Preferred Reporting Items for Systematic Reviews and Meta-Analyses flowchart. The figure depicts the systematic study-selection process following an initial literature search, which yielded 150 articles. Through de-duplication and a thorough review of titles and abstracts, a refined selection effort identified eight studies. Among these, seven were observational studies and one was a randomized controlled trial.

The initial phase of the literature search yielded a total of 150 articles. Following the elimination of duplicates and a meticulous review of titles and abstracts, a refined selection identified eight studies.^12–19^ Among these, seven were observational studies^12–16,18,19^ and one was an RCT.^17^ The subsequent meta-analysis specifically incorporated studies with a comparative design. The PRISMA diagram shown in **Figure 1** visually represents the search approach. Noteworthy is that the RCT followed a comparative, multicenter, and double-blind design, while all the observational studies adhered to a retrospective cohort design. The average follow-up duration across the included studies was 9 months. The timeline of these publications spanned from 2019–2023.

### Baseline characteristics of the included patients

In this meta-analysis, 20,684 participants were included, with 11,805 individuals (57.07%) assigned to the DOAC group and 8879 individuals (42.9%) allocated to the warfarin group. Nearly half of the study cohort consisted of male participants, representing 11,732 individuals (56.7%) out of 20,684. The participants had an average age of 61.63 ± 9 years. The mean baseline CHA_2_DS_2_-VASc score was 3.03 ± 1.1 points, and the mean baseline HAS-BLED score was 2.92 ± 1.0 points. Notably, the baseline data revealed a significant prevalence of conditions like diabetes mellitus, HTN, heart failure, and hyperlipidemia among the included individuals. A comprehensive overview of the baseline characteristics of the study participants is provided in **Table 1**.

### Quality assessment and publication bias

**Supplementary Figure S1:**
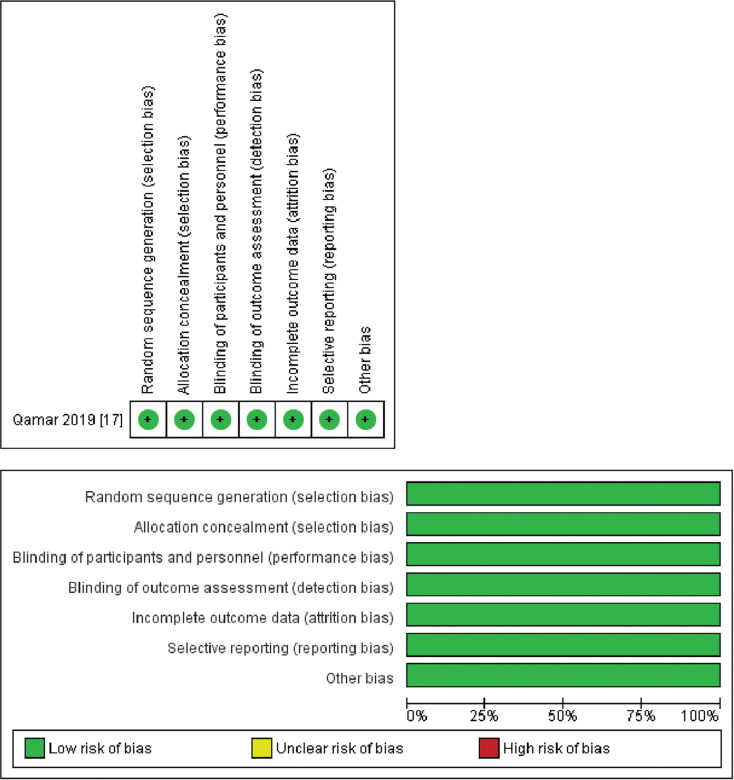
Quality assessment of the included randomized controlled trials.

**Supplementary Figure S2:**
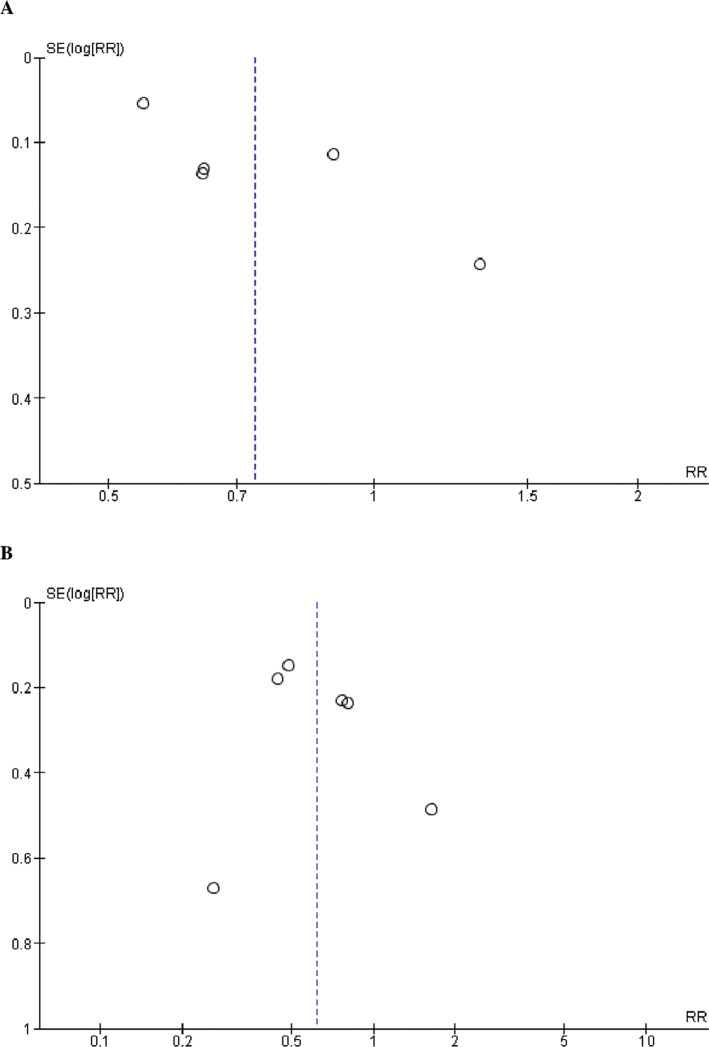
Funnel plots of primary outcomes. Funnel plots for **(A)** all-cause mortality and **(B)** ischemic stroke. Relative risk was used as an effect measure, and standard error was used as a measure of precision. The funnel plots indicated no evidence of publication bias.

Identifying trials with moderate to high quality was achieved using the Cochrane method. This method systematically examines critical elements, such as randomization, allocation concealment, blinding, and outcome reporting, tailored explicitly for scrutinizing RCTs. The detailed findings of this assessment can be found in **Supplementary Figure S1**. Additionally, using the Newcastle–Ottawa scale in the evaluation revealed a spectrum of quality among the observational studies incorporated in the analysis, ranging from fair to good. This systematic tool gauges the quality of non-randomized studies, primarily observational ones, by considering factors such as selection, comparability, and outcome assessment, as delineated in **Supplementary Table S2**. The symmetry observed in the funnel plots assures that the study outcomes remain unaffected by any undue influence of publication bias, as illustrated in **Supplementary Figure S2**.

### Primary outcomes

The primary endpoints encompassed all-cause mortality and ischemic stroke.

#### All-cause mortality

**Figure 2:**
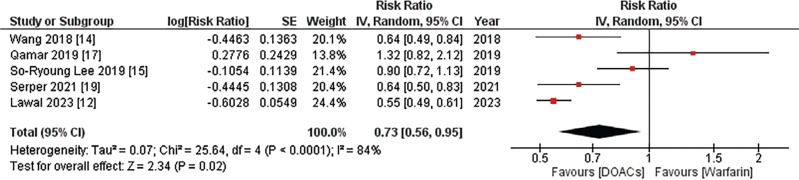
All-cause mortality. The figure presents a meta-analysis incorporating data from five of the eight included studies, revealing a significantly reduced risk of all-cause mortality associated with direct oral anticoagulants compared to warfarin. *Abbreviations:* CI, confidence interval; DOACs, direct oral anticoagulants; IV, inverse variance; RR, risk ratio; SE, standard error.

Information regarding all-cause mortality was provided by five out of the eight studies. The combined analysis indicated that the use of DOACs was linked to a notably reduced risk of all-cause mortality in comparison to warfarin (RR, 0.73; 95% confidence interval [CI], 0.56–0.95; *I*^2^ = 84%; *P* = .02), as depicted in **Figure 2**. Given the high heterogeneity among studies, a sensitivity analysis was conducted, revealing that the exclusion of studies by Wang et al.,^14^ Serper et al.,^19^ and Lawal et al.^12^ individually rendered the results non-significant, with no substantial decrease in study heterogeneity.

#### Ischemic stroke

**Figure 3:**
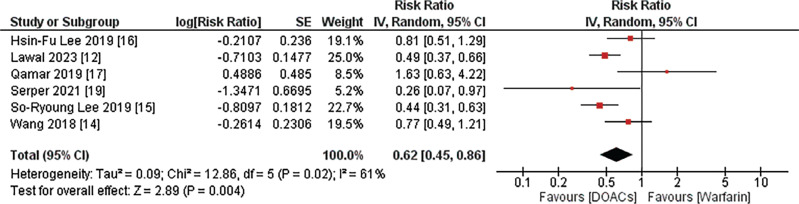
Ischemic stroke. The figure presents a meta-analysis consolidating data from six out of the eight included studies, elucidating a significantly reduced risk of ischemic stroke associated with direct oral anticoagulants compared to warfarin. *Abbreviations:* CI, confidence interval; DOACs, direct oral anticoagulants; IV, inverse variance; RR, risk ratio; SE, standard error.

Data on ischemic stroke were available from six out of the eight studies. The pooled analysis demonstrated that the use of DOACs was correlated with a significantly diminished risk of ischemic stroke compared to warfarin (RR, 0.62; 95% CI, 0.45–0.86; *I*^2^ = 61%; *P* = .004), as illustrated in **Figure 3**.

### Secondary outcomes

The secondary endpoints included major bleeding, GI bleeding, and ICH. A concise summary of the analysis results for these secondary outcomes is presented in **Table 2**.

Data from all eight studies regarding major bleeding were available, and the combined analysis demonstrated a significantly reduced risk of major bleeding associated with DOACs compared to warfarin. For GI bleeding, information from six out of the eight studies was included in the pooled analysis, revealing a non-significant decrease in the risk of GI bleeding with DOACs compared to warfarin. In the case of ICH, data from four out of the eight studies contributed to the pooled analysis, indicating a significantly decreased risk of ICH associated with DOACs compared to warfarin.

## Discussion

AF is the most common arrhythmic illness in adults, with the potential to cause thromboembolism, resulting in severe ischemia events in numerous organs.^20^ Anti-thrombotic therapy is critical in addressing the increased embolic risk, especially for patients with greater embolic risk, according to the CHA_2_DS_2_-VAS_2_ scoring method.^21^ CLD can impair the synthesis of several proteins, including procoagulation and anticoagulation factors, potentially leading to bleeding or thrombosis. As a result, the question arises as to whether AF patients with severe liver disease are at a greater risk of ischemic cerebral stroke or, conversely, of cerebral hemorrhage.^22^ This question creates a significant therapeutic issue in selecting whether to administer anticoagulant medication to these individuals. In the past, the use of DOACs compared to warfarin in patients with AF has proven to be efficacious and safe. However, their usage and comparison have never been undertaken on a broad scale to create universal criteria in patients with AF and CLD.

Our systematic review and meta-analysis of eight studies, which included 20,684 individuals, compared the safety and efficacy of DOACs to warfarin in patients with AF and CLD. Our primary outcomes were all-cause mortality and ischemic stroke, with additional secondary events including major bleeding, GI bleeding, and ICH. In comparison to warfarin, DOACs had a significantly lower risk of all-cause death and ischemic stroke. These findings contradict a previous study by Camm et al.,^22^ which found that, after adjusting for DOAC dosages, overdosage and underdosage were associated with an increased risk of all-cause mortality. However, this increased risk did not result in a significantly greater risk of stroke or systemic embolism. As expected, underdosing was associated with a much lower incidence of bleeding. The surplus of deaths was primarily due to greater rates of cardiovascular mortality from events such as congestive heart failure and myocardial infarction. Overdosage, on the contrary, showed no statistically significant trend toward an increased risk of all-cause death, stroke/systemic embolism, or hemorrhage. Lee et al.^16^ found identical rates of ischemic stroke in three separate DOAC groups and the warfarin group. However, the time spent within the therapeutic range for warfarin remains unknown. In a separate study by Lee et al.,^15^ concentrating on patients with CLD, both the DOAC and warfarin groups had comparable rates of ischemic stroke, severe bleeding, and all-cause death. It is worth noting that our findings differ from those presented in this investigation. In terms of secondary outcomes, our study found a considerable reduction in the incidence of major bleeding and ICH. While the risk of GI bleeding decreased, the difference was not statistically significant. A study conducted by Semmler et al.^23^ found that variations in major bleeding occurrences could be attributable to different indications for DOAC treatment and the type of DOAC used. Mort et al.^24^ observed in their study that 21% of patients with decompensated cirrhosis quit DOAC medication due to actual or perceived bleeding. In total, around one-third of their patients experienced bleeding. While we did not investigate or compare the incidence of thromboembolic events among patients, our findings on bleeding episodes contradict previous research. Contrary to widespread assumption, DOACs were not associated with an increased frequency of GI bleeding. Furthermore, dabigatran and apixaban therapies have resulted in a much-reduced total bleeding frequency compared to other DOACs, as reported in the literature.^25^

Our study has both strengths and limitations. In terms of strengths, a large proportion of our findings were statistically significant, and we found little publication bias across the dataset. However, it is critical to recognize some limits of our investigation. The high proportion of retrospective cohort studies in our analysis reflects the scarcity of RCTs on this particular topic. Future studies focusing on RCTs could improve the robustness and generalizability of our findings. Furthermore, due to the inherent heterogeneity in the available data, our study did not categorize patient data by DOAC type. As a result, our study revealed greater levels of heterogeneity. The potential influence of elevated liver function test results on the safety and efficacy of DOACs and the applicability of our findings to patients with advanced liver disease are also duly recognized. The inclusion of patients with CLD in our study may not provide a comprehensive representation of individuals with more severe hepatic impairment. It is essential to acknowledge the need for caution when considering the administration of DOACs to patients with significantly elevated liver function test results or class B/C cirrhosis, as there may be potential differences in drug clearance and metabolism. Subsequent investigations should strive to rectify these constraints and offer individualized recommendations regarding the use of anticoagulants in patients diagnosed with CLD.

## Conclusion

In conclusion, DOACs have better safety and effectiveness profiles than warfarin, as shown by lower rates of all-cause death, ischemic stroke, severe bleeding, and cerebral hemorrhage. Despite these encouraging findings, it is critical to emphasize the need for additional randomized controlled studies in the future. Such trials would be critical in substantiating and strengthening the databases for DOACs’ comparative efficacy and safety in a wide range of patient populations.

## Figures and Tables

**Table 1:** Baseline Characteristics of the Included Studies

Author (Year)	Study Design	Duration	Total No. of Patients	No. of Patients		Mean ± SD Age (Years)		Male (%)		Hypertension (%)		Diabetes (%)		Heart Failure (%)	
				DOACs	Warfarin	DOACs	Warfarin	DOACs	Warfarin	DOACs	Warfarin	DOACs	Warfarin	DOACs	Warfarin
Lawal et al. (2023)^12^	Retrospective cohort study	2011–2017	10,209	5788	4421	70.3 ± 10.6	72.2 ± 10	57.1	57	87.4	88.2	44.2	47.8	42.3	51
Goriacko and Veltri (2018)^13^	Retrospective cohort study	2009–2016	233	75	158	66 ± 3.5	65 ± 3.37	57.3	59.5	NM	NM	NM	NM	NM	NM
Wang et al. (2018)^14^	Retrospective cohort study	2009–2016	736	342	394	77.3 ± 6.9	77.3 ± 6.9	51	51	69.2	69.2	30.5	30.5	50.9	50.9
Lee et al. (2019)^15^	Retrospective cohort study	NM	4942	3115	1827	67.9 ± 10.2	68.1 ± 10.8	61.8	61.2	70.5	71.1	26.4	27.2	33.3	33.6
Lee et al. (2019)^16^	Retrospective cohort study	2012–2016	2428	1438	990	74.35 ± 10.50	69.9 ± 12.42	62.38	65.56	86.37	80.91	46.31	44.55	20.51	25.45
Qamar et al. (2019)^17^	RCT	NM	1083	718	365	68.4 ± 9.7	68.4 ± 9.7	62.8	67.6	94.1	34	41.2	35.9	63.2	31.5
Yoo et al. (2022)^18^	Retrospective cohort study	2012–2018	382	128	110	70.4 ± 2.87	65.2 ± 3.52	80.5	75.5	53.1	41.8	31.2	36.4	19.5	12.7
Serper et al. (2021)^19^	Retrospective cohort study	2012–2018	815	201	614	64 ± 7.7	64.6 ± 7.5	99.5	98.2	96	98.2	55.2	55.2	10	12.7

Abbreviations: DOACs, direct oral anticoagulants; NM, not mentioned; RCT, randomized controlled trial; SD, standard deviation.

**Table 2:** Secondary Outcomes

Outcome	RR	95% CI	*P* Value	*I* ^2^
Major bleeding	0.64	0.51–0.80	.001	51%
GI bleeding	0.76	0.52–1.11	.15	73%
ICH	0.53	0.35–0.81	.003	0%

*Abbreviations:* CI, confidence interval; GI, gastrointestinal; *I*^2^, heterogeneity; ICH, intracranial hemorrhage; RR, relative risk.

**Supplementary Table S1:** Search Strategy

Database	Search Strategy	Results
PubMed	(((“direct”[All Fields] OR “directed”[All Fields] OR “directing”[All Fields] OR “direction”[All Fields] OR “directional”[All Fields] OR “directions”[All Fields] OR “directivities”[All Fields] OR “directivity”[All Fields] OR “directs”[All Fields]) AND (“mouth”[MeSH Terms] OR “mouth”[All Fields] OR “oral”[All Fields]) AND (“anticoagulants”[Pharmacological Action] OR “anticoagulants”[MeSH Terms] OR “anticoagulants”[All Fields] OR “anticoagulant”[All Fields] OR “anticoagulate”[All Fields] OR “anticoagulated”[All Fields] OR “anticoagulating”[All Fields] OR “anticoagulation”[All Fields] OR “anticoagulations”[All Fields] OR “anticoagulative”[All Fields])) OR “DOACs”[All Fields] OR (“apixaban”[Supplementary Concept] OR “apixaban”[All Fields] OR “apixaban s”[All Fields]) OR (“edoxaban”[Supplementary Concept] OR “edoxaban”[All Fields]) OR (“dabigatran”[MeSH Terms] OR “dabigatran”[All Fields] OR “dabigatran s”[All Fields])) AND (“warfarin”[MeSH Terms] OR “warfarin”[All Fields] OR “warfarin s”[All Fields] OR “warfarinization”[All Fields] OR “warfarinized”[All Fields] OR “warfarins”[All Fields] OR (“warfarin”[MeSH Terms] OR “warfarin”[All Fields] OR “coumadin”[All Fields] OR “warfarin s”[All Fields] OR “warfarinization”[All Fields] OR “warfarinized”[All Fields] OR “warfarins”[All Fields])) AND (“atrial flutter”[MeSH Terms] OR (“atrial”[All Fields] AND “flutter”[All Fields]) OR “atrial flutter”[All Fields] OR (“atrial fibrillation”[MeSH Terms] OR (“atrial”[All Fields] AND “fibrillation”[All Fields]) OR “atrial fibrillation”[All Fields])) AND (“liver cirrhosis”[MeSH Terms] OR (“liver”[All Fields] AND “cirrhosis”[All Fields]) OR “liver cirrhosis”[All Fields] OR (“chronic”[All Fields] AND (“liver diseases”[MeSH Terms] OR (“liver”[All Fields] AND “diseases”[All Fields]) OR “liver diseases”[All Fields] OR (“liver”[All Fields] AND “disease”[All Fields]) OR “liver disease”[All Fields])) OR (“clin liver dis hoboken”[Journal] OR “cld”[All Fields]))	75
Cochrane Library	(Direct oral anticoagulants OR DOACs OR Apixaban OR Edoxaban OR Dabigatran) AND (Warfarin OR Coumadin) AND (Atrial flutter OR Atrial fibrillation) AND (Liver cirrhosis OR Chronic liver disease OR CLD)	50
Google Scholar	(Direct oral anticoagulants OR DOACs OR Apixaban OR Edoxaban OR Dabigatran) AND (Warfarin OR Coumadin) AND (Atrial flutter OR Atrial fibrillation) AND (Liver cirrhosis OR Chronic liver disease OR CLD)	25

*Abbreviation:* MeSH, Medical Subject Heading.

**Supplementary Table S2:** The Newcastle–Ottawa Scale

Study	Selection				Comparability	Outcomes			Total
	Representativeness of the Exposed Cohort	Selection of the Non-exposed Cohort	Ascertainment of Exposure	Demonstration That Outcome of Interest Was Not Present at Start of Study	Comparability of Cohorts on the Basis of the Design or Analysis	Assessment of Outcome	Was Follow-up Long Enough for Outcomes to Occur	Adequacy of Follow-up of Cohorts	
Lawal et al. (2023)^12^	*	*	*	*	**	*	*	*	*********
Goriacko and Veltri (2018)^13^	*	*	*	*	*	*	*	*	********
Wang et al. (2018)^14^	*	*	*	*	*	*	*	*	********
Lee et al. (2019)^15^	*	*	*	-	*	*	*	*	******
Lee et al. (2019)^16^	*	*	*	*	**	*	*	*	*********
Yoo et al. (2022)^18^	*	*	*	*	*	*	*	*	********
Serper et al. (2021)^19^	*	*	*	*	*	*	*	*	********

This table thoroughly assesses the quality of observational studies incorporated into the meta-analysis, using the Newcastle–Ottawa scale. The evaluation delineates a spectrum of study quality, ranging from fair to good. In the Newcastle–Ottawa scale, an asterisk (*) signifies a point awarded for meeting specific criteria, with good quality defined as 3 or 4 stars in the selection domain AND 1 or 2 star(s) in the comparability domain AND 2 or 3 stars in the outcome/exposure domain. Fair quality is characterized by 2 stars in the selection domain, 1 OR 2 star(s) in the comparability domain, AND 2 or 3 stars in the outcome/exposure domain. Poor quality is indicated by 0 or 1 star(s) in the selection domain OR 0 stars in the comparability domain OR 0 or 1 star(s) in the outcome/exposure domain.
